# Hybrid Fabrics for Ohmic Heating Applications

**DOI:** 10.3390/polym17101339

**Published:** 2025-05-14

**Authors:** Jiří Militký, Karel Kupka, Veronika Tunáková, Mohanapriya Venkataraman

**Affiliations:** 1 Department of Material Engineering, Faculty of Textile Engineering, Technical University Liberec, 461 17 Liberec, Czech Republic; jiri.militky@tul.cz (J.M.); veronika.tunakova@tul.cz (V.T.); 2 TriloByte Statistical Software, 533 52 Pardubice, Czech Republic; kupka@trilobyte.cz

**Keywords:** hybrid fabric, SS fibers, electrical properties, Joule heating, relation between voltage and current, surface temperature

## Abstract

Textile structures with ohmic (Joule) heating capability are frequently used for personal thermal management by tuning fluctuations in human body temperature that arise due to climatic changes or for medical applications as electrotherapy. They are constructed from electrically conductive textile structures prepared in different ways, e.g., from metallic yarns, conductive polymers, conductive coatings, etc. In comparison with other types of flexible ohmic heaters, these structures should be corrosion resistant, air permeable, and comfortable. They should not loose ohmic heating efficiency due to frequent intensive washing and maintenance. In this study, the basic electrical properties of a conductive fabric composed of a polyester/cotton fiber mixture and a small amount of fine stainless-steel staple fibers (SS) were evaluated and predicted. Even though the basic conductive component of SS fibers is iron and its electrical characteristics obey Ohm’s law, the electrical behavior of the prepared fabric was highly nonlinear, resembling a more complex response than that of a classical conductor. The non-linear behavior was probably due to non-ideal, poorly defined random interfaces between individual short SS fibers. A significant time–dynamics relationship was also shown. Using the Stefan–Boltzmann law describing radiation power, we demonstrated that it is possible to predict surface temperature due to the ohmic heating of a fabric related to the input electrical power. Significant local temperature variations in the heated hybrid fabric in both main directions (warp and weft) were identified.

## 1. Introduction

Electrical resistivity *r* [S^−1^ m = Ω.m] can be used for the rough classification of substances into conductors (*r* = 10^–8^ Ω.m for majority of metals till 10^–5^ Ω.m for carbons), semi-conductors (*r* = 10^–5^ Ω.m–100 Ω.m), and insulators (*r* = 100 Ω.m–10^16^ Ω.m). Most synthetic fibers are electrical insulators with *r* = 10^12^ Ω.m–10^14^ Ω.m. Antistatic fibers have *r* = 10^7^ Ω.m–10^10^ Ω.m. For materials designed to shield the electromagnetic field, an *r* of less than 10^3^ Ω.m is required. Electrically conductive fibers have an *r* of about 10^–7^ Ω.m or less [1,2,3].

The electrical resistivity of textile fibers is directly connected with their hydrophilicity, which is the reason that natural fibers (*r* = 10^5^ Ω.m–10^7^ Ω.m) dissipate electrical charge in atmospheric conditions with some degree of humidity.

Electrical conductivity is, therefore, a crucial parameter in terms of reducing the tendency to accumulate electrostatic charge (anti-static properties), improving resistance to electromagnetic radiation (EMI shielding), and designing smart textiles containing conductive paths for the implementation of clothing electronics and sensors. Electrically conductive textiles are often used in special clothing and technical textiles, where it is possible to partially replace classical metals or other materials using easily formable (textile) structures [1].

Electrically conductive textiles can be made from yarns containing electrically conductive fibers made of metals (hybrid yarns) [4], carbon structures [5], textiles containing conductive nanoparticles, or intrinsically conductive polymers [1,2,6,7,8,9]. Very often, conductive coatings based on conductive particles or preferably nanoparticles are also used [10]. It is common for all kinds of electrically conductive textiles from hybrid yarns to have a structure resulting from classical textile technologies, such as spinning, weaving, knitting, and nonwoven technologies, which are used for their preparation [9].

Cold drawing (for ductile metals) and hot drawing (for brittle metals) through conical holes are used to produce wires up to 100 μm only; therefore, special techniques such as the Taylor process or bundle drawing should be used [3]. Instead of metal fibers, metal-coated plastics or metal surface layers covering whole fibers are also frequently used [11,12].

Another option for producing electrically conductive textiles is to coat them with conductive polymers or impregnate them with conductive organic or inorganic particles.

Conductive polymers are generally characterized by a conjugated bond system with π-electrons [1,2]. To ensure conductivity, it is usually necessary to perform partial oxidation or reduction. These polymers are often infusible and have a high surface tension (more than 150 mN m^−1^). They are, therefore, mostly used in the form of dispersions unless they are formed by polymerization in situ on the surface of materials. Using a suitable dispersion medium, the electrical resistivity of polyaniline can be reduced from the original 10^−3^ Ω.m to 10^−5^ Ω.m [3].

The electrical conductivity of conductive polymers differs greatly from that of metals, where there is no band gap and free electrons are present in the conduction band at ambient temperatures. In semiconductors, the band gap is narrow, so their conductivity is between that of metals and insulators. The degree of electrical conductivity of polymers also depends on the degree of chain ordering and is, therefore, lower than that of inorganic semiconductors. The differences between charge transfer by free electrons and transfer by jumps of different lengths are evident, for example, in the difference in the dependence of electrical conductivity on temperature [1,2,3]. For metal conductors, electrical conductivity is a decreasing function of temperature, whereas, for semiconductors and conductive polymers, electrical conductivity is an increasing function of temperature. The selection of suitable methods for adding conductivity to textiles depends on the requirements, such as durability, washability, air permeability, and comfort, linked to the application aims. The hybrid conductive textiles created from hybrid staple yarns containing a small percentage of fine staple metal fibers are a good candidate for applications connected with clothing or textiles that are in direct contact with humans [4,6,13,14,15].

Stainless steel with enhanced corrosion protection in the form of thin and short wires (typically in the range of 2.5–5 cm) has a wide range of applications in textiles. Very fine iron-based alloys (also containing nickel and chromium) in the form of staple SS fibers produced, e.g., by company Bekaert from Zwevegem, Belgium under the name Bekinox, are intended for application in the textiles industry as part of a mixture with other textile fibers. This kind of fiber is widely used for the creation of textile structures with enhanced electrical conductivity, EMI shielding, and anti-static properties [4,14,16]. Mixing with other fibers in yarns is beneficial if the SS fibers’ diameter is sufficiently small (6–8 µm), ensuring sufficient flexibility. Optimized techniques for the creation of yarns and fabrics based on these fibers already exist [9]. Therefore, the fiber Bekinox was here selected as a representative of industrially produced material for this research.

The main aim of this study is to investigate the ohmic heating of hybrid fabrics including variations in their temperature profiles. A woven fabric composed of hybrid yarns with a small percentage of extra fine staple stainless-steel fibers was used to test the basic electrical properties and Joule heating. The electrical resistance of the hybrid fabric is predicted using a simple model of an infinite regular grid with nodes simulating real fabric. The nonlinear relation between voltage and current (no validation of Ohm’s law) is experimentally evaluated. The dependence of voltage and electrical resistance on the length between jaws (clamps) is modeled by the exponential type function. The influence of input electrical power on surface temperature of hybrid fabric is predicted via the relationship based on the Stefan–Boltzmann law.

## 2. Principle of Ohmic Heating

Many studies present a comprehensive review of ohmic heating applications in various areas [15,17,18]. Textile structures with ohmic (Joule) heating capabilities are often used for personal thermal management by tuning the fluctuations in human body temperature that arise due to climatic changes. These structures are also useful in biomedical and cosmetic applications (e.g., thermotherapy) [10].

If substances are placed in an electric field, either the transport of electrically charged particles can occur (i.e., an electric current is generated accompanied by energy dissipation), which is typical for conductors, or dipoles and induced dipoles are formed, which is accompanied by energy accumulation (this process is reversible and typical for insulators).

During the ohmic heating of conductors’ electrical power, *P* [W] is delivered to a conductive material, dissipated and completely transformed into heat. Electrical power is defined as product of voltage *V* [V or J/C] and current *I* [A,], i.e., $P=V^{.}I$.

It is well known that the temperature dependence of electrical resistance *R* [Ω] of metallic conductive materials (the electrical resistance of the metallic conductive materials is governed by the collision process) is in the wide range, approximately linear, and can be described as follows [17]:

(1) $$R\left(T\right)=R_{0}\left[1+\alpha_{T}\left(T-T_{0}\right)\right]$$

where *T*_0_ is the reference temperature (usually 20 °C), and $\alpha_{T}$ is the temperature coefficient of resistivity (unit (°C)^−1^). For iron, *α =* 6.41 × 10^−3^ (°C)^−1^. Under the validity of Ohm’s law (*V* = *R.I*), it is then possible to express the relation between input electrical power *P* and generated temperature in conductors in the form:

(2) $$T=T_{0}+\left[\frac{P}{I^{2}.R_{0}}-1\right]/\alpha_{T}$$

These relations are valid for conductive metallic materials.

Zhang et al. [19] prepared a highly stretchable electrically driven heater based on electrically conductive carbonized weft-knitted fabrics. They found that the temperature of the heater is linearly dependent on the power density and square of input voltage, which follows the assumption of Ohm’s law (i.e., a linear relation between the voltage and current). Based on the assumption that, in equilibrium, the input Joule heat will be equal to the heat loss through convection, they proposed a differential equation for the dynamics of heating. The temperature of the heater according to this model is linearly related to the input power density (*V.I*) and inversely related to the convective heat-transfer coefficient (*h*).

Hybrid conductive textiles are porous structures with porosity typically over 50% (yarns) or 75% (fabrics); they contain a high portion of nonconductive fibers. There are three main problems with these structures, addressed using Equation (2).

The first problem is the assumption of validity of Ohm’s law (i.e., current vs. voltage dependence should be linear), which is an overly ideal assumption. It was found that the current–voltage curves (*I*–*V* curves) of the conductive fabric coated with one to five layers of graphene are slightly convex and increasing [20]. The *I*–*V* curves of glass-fiber-reinforced graphite/carbon black/polyethersulfone (G/CB/PES) composites were highly nonlinear [21]. When these composites were powered at a low voltage, the current was linearly increased with a constant slope (according to Ohm’s law). With increasing voltage, the slope of *I–V* dependence was lowered and, with further increases in voltage, the slope again increased.

We found that accelerating the oxidation rate of some metals and thickening the metal oxide layer due to increases in the current can increase the electrical contact resistance and change the shape of *I–V* curves [22]. The nonlinear course of the *I*–*V* curves for textile materials containing SS fibers were also observed in a previous study [23]. It is therefore more suitable to use the relation $I=f\left(V\right)$, where the function $f\left(V\right)$ should be evaluated based on experimental data using a suitable regression-type model [24].

The second problem is the identification of right value of *R*_0_, which is generally the sum of the bulk resistance *R*_b_ of the material and the contact resistance due to the contacts at the binding points *R*_c_. Usually, it is apparent that, due to their asperities, the contact area of the contacting surfaces is very small. The contact resistance is calculated according to the Holm relation [25]:

(3) $$R_{c}=\frac{C}{2.n}\sqrt{\frac{\pi .H}{F}}≃\frac{1}{n}\sqrt{\frac{1}{F}}$$

where *C* [Ω.m] is electrical resistivity, *n* is the number of contacting points, *H* [N/m^2^] is hardness, and *F* [N] is the force applied in contact points. Contact resistance may be significant for these reasons [16,26]: (a) the surfaces in contact are not always flat and smooth, (b) the surfaces in contact are not always ideally clean, and they often contain natural oxide and other surface contaminants, and (c) the contact resistance strongly depends on the force.

The contact resistance of stainless-steel yarns passivated by a thin layer of Cr_2_O_3_ on the surface obeys nonlinear electrical behavior [23]. A simple method for eliminating contact resistance problems in hybrid yarns is based on the measurement of two or more sections of the yarns [27].

**Figure 1:**
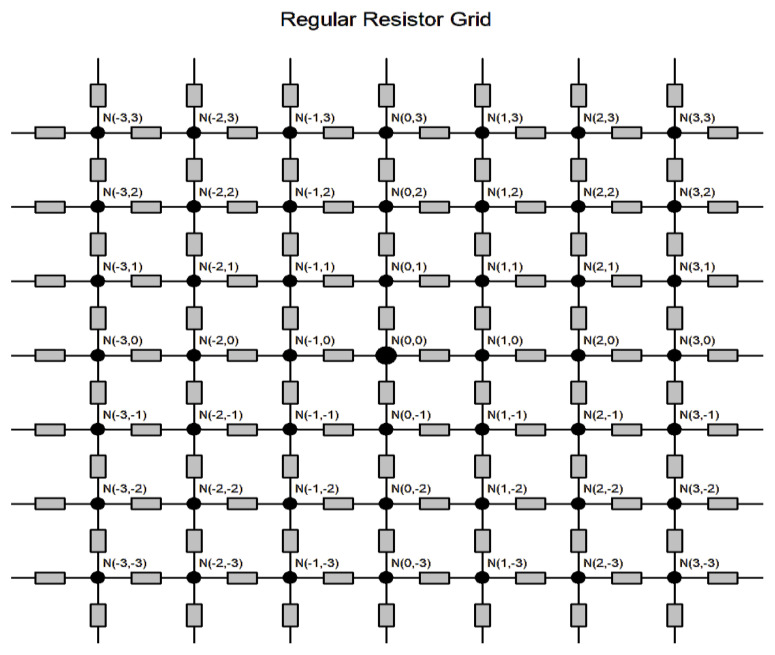
Infinite regular grid simulating a real fabric with nodes N(*i*, *j*).

The third problem is connected with the construction of textile structures composed from conductive yarns where the contact points (nodes) N(i, j) act as resistors [28]. For the purpose of the simulation, a regular grid (see Figure 1) acting as a semiconductor (or varistor) rather than a passive resistor is used.

This manifests as a non-linear current–voltage characteristic [29,30]. To calculate the resistance between the origin and the point (*i*, *j*) in an infinite square grid, the lattice Green’s function is frequently used [30]. The solution for this case has the following form [31]:

(4) $$R_{0}\left(i,j\right)=\frac{1}{\pi }\int_{0}^{\pi }\frac{\left[1-\exp\left(-\left|i.\alpha \right|\right).\cos\left(j.\beta \right)\right]}{\sinh\left|\alpha \right|}\;d\beta$$

where α and β are interrelated variables of integral representation: see Equation (4). For $0<\beta <2^{.}\pi$, there is a complex relation between α and β (i is the imaginary unit)

(5) $$\alpha =i^{.}\log\left[2-\cos\beta +\sqrt{3-4.\cos\beta +\cos^{2}\beta }\right]$$

The program used in Mathematica for the solution of Equation (4) is given in [30]. For large values of *i* or/and *j*, the approximate equation is valid [32]:

(6) $$R_{0}\left(i,j\right)=\frac{R}{\pi }\left(\ln\left(\sqrt{i^{2}+j^{2}}\right)+\gamma +\frac{\ln\left(8\right)}{2}\right)$$

where γ=0.5772 is the Euler–Mascheroni constant and *R* is the resistance of one node (assumed to be the same for all nodes). It is valid that, for an increasing value of *j* (corresponding to the clamp distance in the y direction), the resistance of the infinite square grid is concave and growing. The results of the calculations presented in [30] show that, from a distance of eight nodes (relative resistance $R_{0}\left(i,j\right)/R$ about 1.17), there is an increase in relative resistance that increases almost linearly, with a slope around 0.03. For a regular woven textile with sett *S* yarn per cm, the origin is at *S*/2 and number of nodes is *S*. For the longer textile pieces, there is thus a significant increase in resistance due to number of contact points between weft and warp yarns; there is also a case of low resistance of one contact *R*. Replacing real textiles with an infinite regular grid is acceptable for cases when nodes are created from conductive monofilaments, i.e., binding points are conductors.

For hybrid fabrics (where weft and warp yarns are composed from staple fibers containing only a small percentage of conductive SS fibers), conductive contact points are not regular and not equal to binding points. There are frequently nonconductive contacts between weft and warp yarns (i.e., from resistors). Such contacts may also exist along both single yarns and create high-resistivity spots. A long single yarn is typically not very conductive, as the short *SS* wires in the yarn structure may not be connected. An applied electrical current in the fabric will then spread over the grid randomly and result in accumulating charge at the interface and eventually through the interface.

## 3. Materials and Methods

### 3.1. Materials

The conductive materials provided by Sintex, Česká Třebová, Czech Republic were hybrid yarns of fineness 30 tex containing fine stainless steel (SS) metal fibers (Bekinox) of diameter 8 µm and with an average length of 45 mm. The aspect ratio (length/diameter ratio, *l/d*) of the SS is 562.

The hybrid yarns composed of conventional polyester fiber (59 wt%), cotton fiber (31 wt%), and Bekinox stainless steel fibers (10 wt%) were used. These components were mixed at the drawing frame. Yarns were produced using ring-spinning technology.

**Figure 2:**
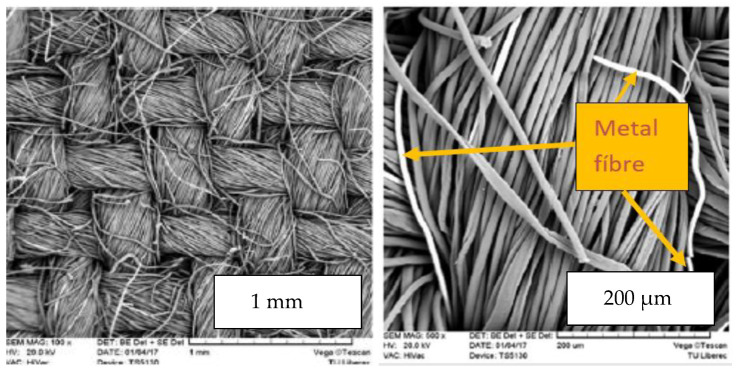
Hybrid fabric images on different scales.

We produced a twill 2/1 weave hybrid fabric (warp sett 39 cm^−1^, weft sett 22 cm^−1^) with a fabric thickness *t* = 0.36 mm and a planar mass *w*_f_ =190 g m^−2^. A percentage of stainless steel close to the percolation threshold of the conductive component ensures electrical conductivity. A small percentage of steel fibers does not significantly change the processability and comfort of the yarns and fabric. Fabric images in different scales are shown in Figure 2.

### 3.2. Measurements

The element composition of the Bekinox fibers was examined using a Zeiss Ultra Plus scanning electron microscope (Zeiss, Oberkochen, Germany) with an Oxford X-max 20 energy dispersive X-ray spectrometer (Oxford Instruments, Abingdon, UK).

**Figure 3:**
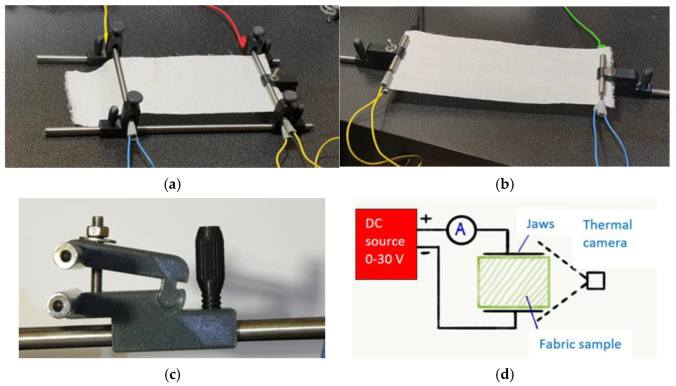
(**a**) Wide measuring jaws, (**b**) narrow measuring jaws, (**c**) detail of measuring jaws, (**d**) measurement setup scheme.

The electric properties and temperature generated by ohmic heating were measured on a custom-made stand. The measuring stand was composed of wide 4-electrode stainless steel measuring jaws adjustable in 2 axes (A), narrow 4-electrode stainless steel measuring jaws adjustable in 1 axis (B, C), an ammeter (range 0–20 A), a DC power source (working ranges 0–30 V, 0–30 A) and a FLIR E4 thermal camera (see Figure 3). Stainless steel 10 mm circular-cross-section rods were used as measuring jaws with an applied force of 100 N. The upper and lower jaws were electrically connected. This measurement set up was used to measure the ohmic heating and surface temperature of hybrid fabrics. The intensity of ohmic heating was tuned by changing the voltage and current.

**Software Development:** The authors developed the analytical software, which is available from them, in the DARWin language for the QCExpert version 2 software system.

## 4. Results and Discussion

### 4.1. Characterization of Materials

**Figure 4:**
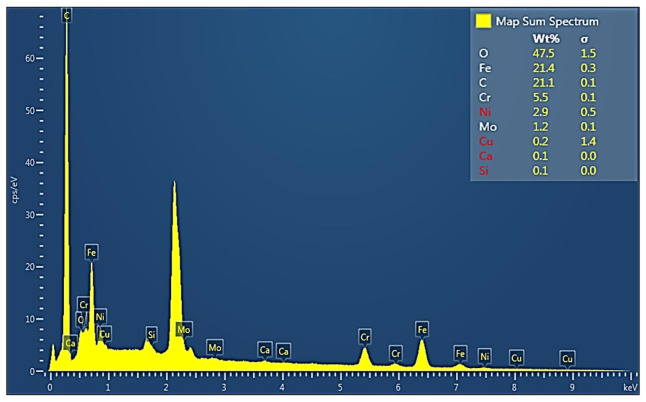
EDS spectrum of the Bekinox fibers’ surface.

The elements on the surface of the Bekinox fibers were identified via EDX analysis (see Figure 4). A detailed interpretation of the EDX spectra is given in [33].

**Figure 5:**
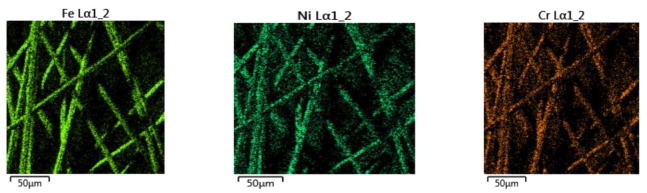
Spectral maps of the most common atoms on the Bekinox fibers’ surface.

Most abundant is oxygen, which corresponds with the assumption that some metals on the surface of Bekinox (in the chrome–nickel alloy of iron) can also be found in the form of oxides. The spectral maps of the three most common atoms on the Bekinox fibers’ surface are shown in Figure 5.

It is clear that the Bekinox fibers’ surface is composed from a mixture of metals and potentially their oxides. The hybrid material density ρ_w_ of the fiber mixture is necessary for the evaluation of the majority of the geometrical and physical properties of hybrid yarns and textiles. The density of the hybrid yarn was calculated from its definition using the weight fractions of the components and their densities as the harmonic mean [3]:

(7) $$\rho_{w}=1/(w_{p}/\rho_{p}+w_{c}/\rho_{c}+w_{b}/\rho_{b})$$

For the polyester component, the mass fraction was *w*_p_ = 0.59 and the density *ρ*_p_ = 1360 kg m^−3^; for the cotton component, the mass fraction was *w*_c_ = 0.31 and density *ρ*_c_ = 1560 kg m^−3^; and, for the Bekinox component, the mass fraction was *w*_b_ = 0.10 and density *ρ*_b_ = 7800 kg m^−3^. By entering these values into Equation (7), it was determined that the hybrid material density was equal to ρ_w_ = 1549.52 kg m^−3^. The equation for the mass of fabric per meter square *W* = ρ_w_ *t*, as recorded in [3], is simply the product of the fabric thickness *t* and the density of the hybrid system ρ_w_ calculated according to Equation (7).

The volume porosity *P_o_* [%] of the investigated fabric was then calculated from the simple equation:

(8) $$P_{o}=100\;\left(1-\frac{\;w_{f}}{t\;\rho_{w}}\right)$$

Based on Equation (8), where *w_f_* is the fabric weight, we calculated the volume porosity P_o_ = 65.94%.

The mass fraction of Bekinox *w*_b_ is much higher than the volume fraction of Bekinox *v*_b_ because the density of Bekinox is very high. Based on the relation derived in [3], *v*_b_ was calculated from the following formula:

(9) $$v_{b}=\frac{w_{b}}{\rho_{b}\left(\frac{w_{p}}{\rho_{p}}+\frac{w_{c}}{\rho_{c}}+\frac{w_{b}}{\rho_{b}}\right)}$$

The volume fraction calculated from Equation (9) is equal to ***v*_b_** = 0.01987, i.e., the volume portion of Bekinox is only 1.98%. This indicates that contact between individual Bekinox fibers is not very frequent.

### 4.2. Electrical Properties of Hybrid Fabrics

For the purposes of ohmic heating, it is necessary to investigate the form of the dependence of current on voltage and the dependence of resistance on the distance between clamps for external powering.

#### 4.2.1. Current–Voltage Measurement

**Figure 6:**
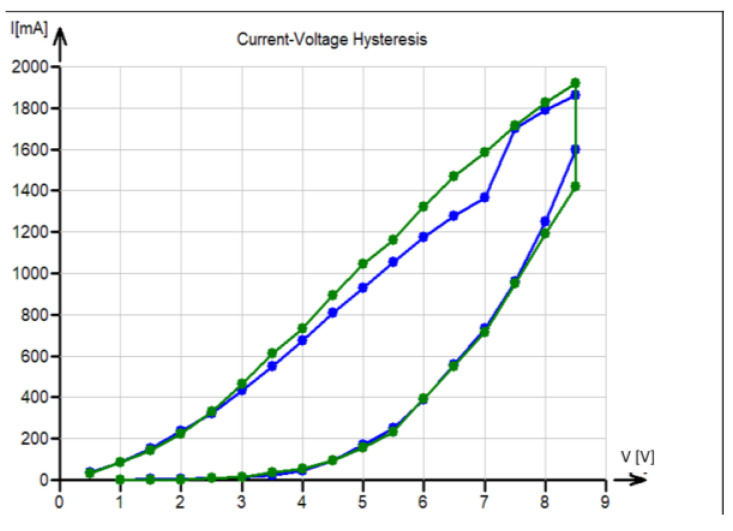
Hysteresis observed in current–V characteristics (green—weft direction, blue—warp direction).

The *I*–*V* curves are essential for checking the validity of Ohm’s law. The experimental response of the current for the range of voltage from 1 V to 10 V is shown in Figure 6. The lower (increasing) branch of curves in Figure 4 was followed by a 10 min relaxation hold at 8.5 V, and then the voltage was decreased back to 0 V (upper branch). These measurements were realized in the warp (blue) direction and the weft (green) direction. It is evident that the curves are far from the line characterizing Ohm’s law, and there are some reasons (probably due to fabric relaxation) for slight changes in the hysteresis curves.

The prediction of ohmic heating based on the relations described in Section 2 cannot, therefore, be fully accepted, and strong hysteresis in current–voltage characteristics must be taken into consideration.

#### 4.2.2. Resistance–Electrical Field Measurement

**Figure 7:**
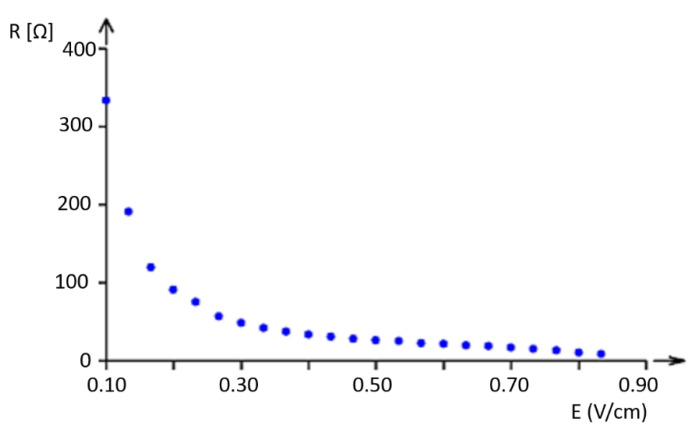
Experimental dependence of resistance on electric field intensity.

**Figure 8:**
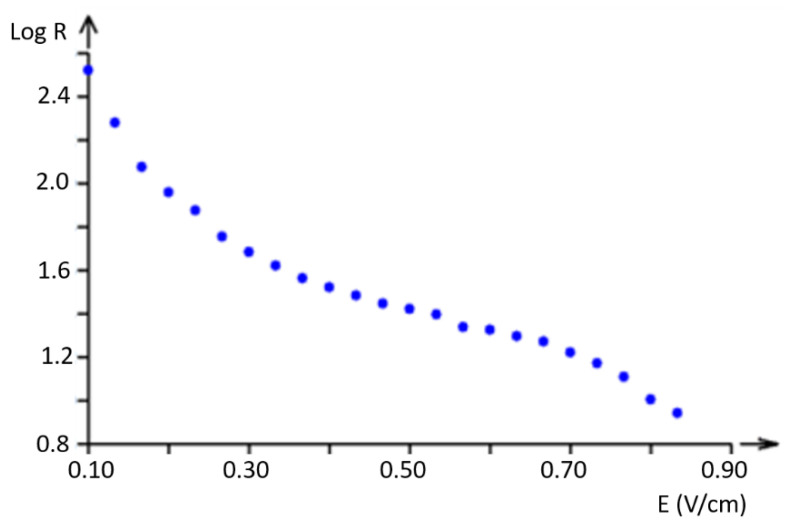
Observed dependence of resistance in the decadic logarithmic scale on electric field intensity.

The experimental dependence of resistance on electric field intensity is shown in Figure 7 and Figure 8.

Resistance was measured in a sample with a square area of 10 cm × 10 cm. The electric field intensity was calculated as E=V/d, where *d* is the distance between the ammeter clamps. For better identification of the shape, the curve was created with resistance in a logarithmic scale (see Figure 8).

It is clear that the decadic logarithmic scale reveals the non-homogeneous behavior of the phenomenon, with a possible change in electrical field intensity (voltage gradient) of about 0.6 V/cm; this demonstrates that the tested samples are not materials that obey Ohm’s law.

#### 4.2.3. Influence of Distance on Electrical Properties

**Figure 9:**
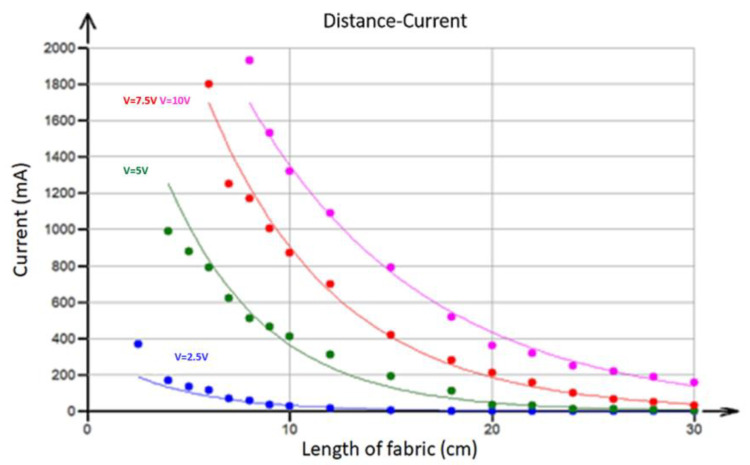
The relationship between current and the distance between electrodes at different voltages. Solid lines are fitted by nonlinear regression curves.

For the design of ohmic heating systems, it is necessary to determine how the distance between the electrodes of the external power source affects the electrical properties. The dependence of the observed current flowing through the fabric (constant width = 12 cm) in the warp direction (in milliamperes) on the distance between electrodes at four different voltages (2.5, 5, 7.5, and 10 V) is shown in Figure 9.

It is clear that, when the distance between the electrodes *L* increases, the current *I* drops nearly exponentially (solid lines). This dependence was approximated using an exponential type of curve (see [24]):

(10) $$I=a\;b^{L}$$

where *a* and *b* are parameters and their estimates were obtained via least squares regression. The estimated parameter values obtained using nonlinear least squares regression are given in Table 1.

Similarly, resistance (calculated using the previous current data by voltage division) depends exponentially on the distance between electrodes. This observation decisively rules out the simple resistor grid model (see Equation (4)), where the dependence is rather logarithmic.

**Figure 10:**
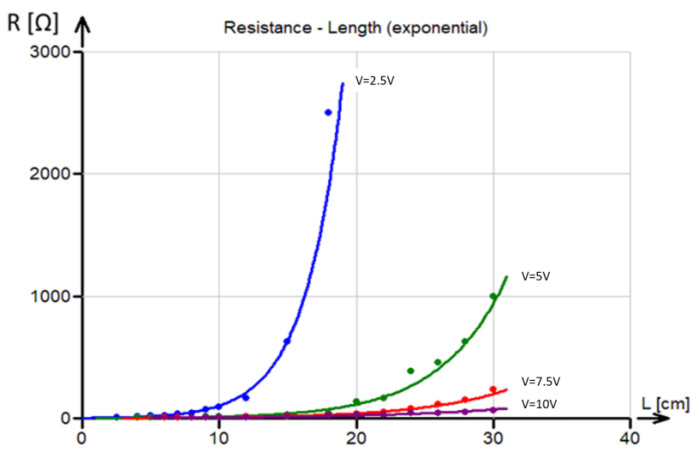
The relation between resistance and the distance between electrodes at different voltages. Solid lines are fitted using nonlinear regression curves.

The dependence of the observed resistance of the fabric (constant width = 12 cm) in the warp direction (in ohms) at four different voltages (2.5, 5, 7.5, and 10 V) is shown in Figure 10.

This dependence was also approximated using an exponential curve (see [24]):

(11) $$R=c\;d^{L}$$

where *c* and *d* are parameters and their estimates were obtained using least squares regression. The estimated parameter values obtained using nonlinear least squares regression are given in Table 2.

### 4.3. Surface Temperature Due to Ohmic Heating

Electrical power (the product of direct current and electrode voltage, *U*.*I*) during ohmic heating was used for the generation of heat, changing the material surface temperature. The temperature of the fabric surface was measured using a FLIR E4 infrared camera. For the prediction of this temperature, we can assume that electrical power energy is converted into heat power, which is emitted and increases the surface temperature of the fabric.

According to the Stefan–Boltzmann law (SBL), a warm body radiates thermal energy. According to Planck’s law, the radiating power is proportional to the fourth power of the body’s temperature. Assuming that most of the heat power emitted from the fabric is equal to electric power *P*, the surface temperature can be expressed by a simple formula:

(12) $$T=\sqrt[{4}]{\frac{P}{S\;k_{S}\;\varepsilon \;\sigma }}-T_{o}$$

where σ is the Stefan–Boltzmann constant, *T* [°C] is the produced surface temperature, ε is emissivity ε≤1 (the emissivity of polyester is between 0.9 and 0.95); *S* is the fabric area (both sides) m^2^; *k_S_* is an empirical correcting factor of effective surface extension due to the fibrous structure (*k_S_* > 1); and *T*_0_ is the absolute temperature offset. The *k*_S_ can be predicted from the surface area of the woven fabrics, calculated using Peirce’s geometrical model [34].

The offset *o* is set to −273 °C. The Stefan–Boltzmann constant is equal to 5.67 10^−8^ Wm^−2^K^−4^.

**Figure 11:**
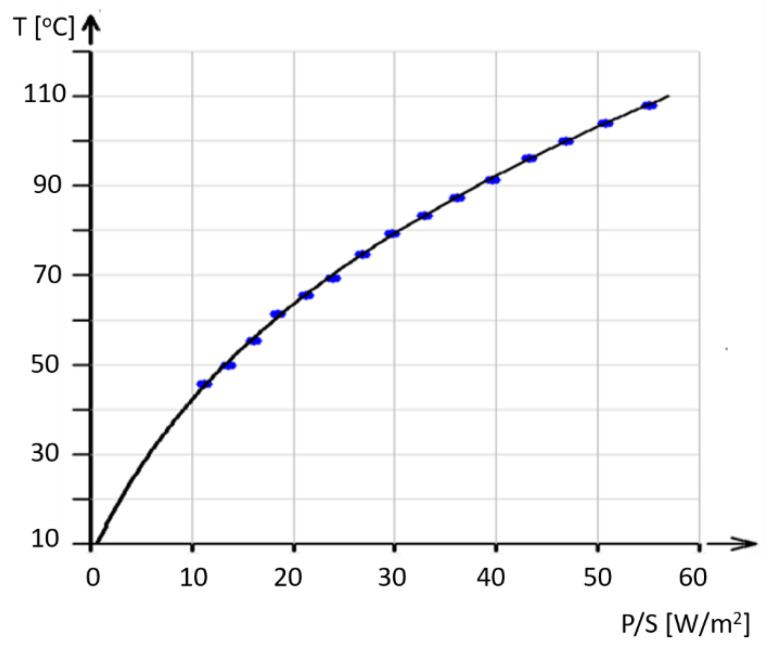
Experimental data (blue points) and curve based on model (12), obtained via nonlinear regression, describing the surface temperature.

Determining the values of *S*, ε, and σ allows us to estimate the effective surface factor *k_S_*. Experimental data and the prediction curve fitted according to Equation (12) using nonlinear regression (see [24]) with *k_S_* as an estimated parameter are shown in Figure 11 (each point is recorded after establishing the equilibrium temperature). The unknown parameter *k_S_* was estimated to be *k_S_* = 5; this suggests that the effective surface of the fabric is 5 m^2^/m^2^.

### 4.4. Heat Profiles

**Figure 12:**
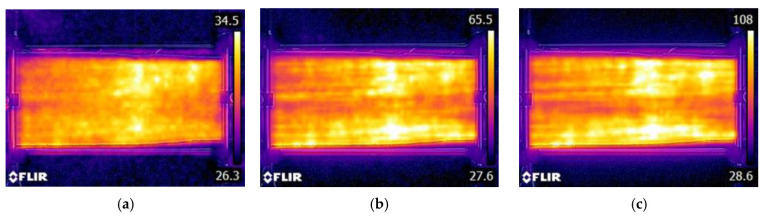
Infrared images at 300 (**a**), 1000 (**b**), and 2000 (**c**) mA with maximal temperatures of 34.5 °C, 65.5 °C, and 108 °C respectively. Sample size 30 cm × 12 cm.

The distribution of temperature in the fabric at different currents is shown in Figure 12. Since the temperature increase at a given place is proportional to the fourth root of power, $\Delta T\propto \sqrt[{4}]{P}$ $P=I^{2.}R$, it follows that higher temperature spots correspond to higher resistance (e.g., due to the lower density or poor connection of the *SS* wires).

**Figure 13:**
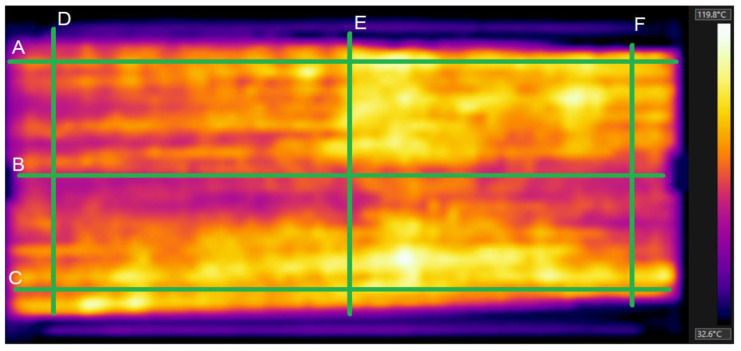
Infrared image of the electrically heated fabric.

Since the temperature distribution in the heated fabric is rather uneven, the average temperature was used as the first approximation. However, a surface integral would be more appropriate for a rigorous approach. The measured temperature profiles are shown in Figure 13.

**Figure 14:**
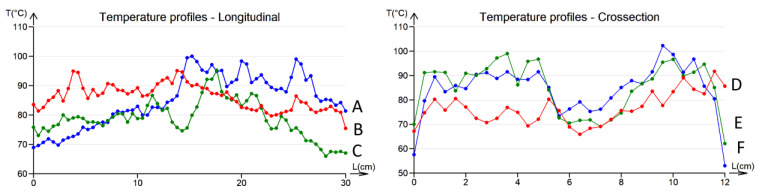
Infrared image of the electrically heated fabric and selected temperature profiles along horizontal (A–C) and vertical (D–F) green lines.

Figure 13 was obtained using a setup to measure the hybrid fabrics’ ohmic heating (see Figure 3d), where electrodes were clamped on the left and right edges of the sample. Selected temperature profiles at selected positions A, B, and C in the longitudinal directions and D, E, and F in the cross directions are shown in Figure 14.

The graphs of the temperature profiles correspond to position lines A, B, C, D, E, and F in the thermal images (see Figure 10) and are labeled accordingly. The relatively large variations in temperatures in the range from approximately 60 °C to 100 °C have the following causes:

✓ The structure of the fabric (twill 2/1 weave) and the structure of the staple hybrid yarns, which are responsible for the large total porosity.
✓ The random variations in the electrical properties (apparent electrical resistance) of hybrid yarns.

These temperature variations are not limited to the proper utilization of these fabrics. The maximum temperature of ohmic heating should be below the softening point of other fibers. Mixtures with PP fibers could also be used, where 100 °C is near the maximum acceptable level.

## 5. Conclusions

The basic reasons for the decrease in the textile fibrous materials’ electrical resistivity were briefly discussed. The peculiarities of hybrid materials partially composed of fine staple stainless steel fibers as active substances were demonstrated. Conductive fabrics made of hybrid yarns were characterized. We found that the behavior of fabrics made from hybrid yarns does not follow Ohm’s law and is highly nonlinear. The infinite regular grid model simulating real fabric geometry was discussed.

The dependence of current flowing through the fabric on the distance between electrodes at four different voltages, 2.5, 5, 7.5, and 10 V, was modeled using exponential curves.

We predicted and measured the surface temperatures in fabric made from hybrid yarns with a small percentage of stainless-steel Bekinox fibers at different currents.

It was found that the temperature fluctuations across the fabric’s surfaces are caused by higher resistance (e.g., due to lower local densities or poor connection of the *SS* fibers). The main results are as follows:

- The evaluation of nonlinear dependence between current and voltage;
- The predicted dependence of current and voltage on the distance between electrodes;
- An evaluation of the surface temperature of ohmically heated hybrid fabrics as a function of input electrical power.

This work shows that classical analysis based on the a priori assumption of the validity of Ohm’s law cannot be directly used to describe the electrical characteristics of more complicated hybrid textile structures. On the other hand, these structures are beneficial for realizing the production of ohmic heating textile apparel due to their durability and comfort.

## Figures and Tables

**Table 1:** Parameter estimates of Equation (10).

Voltage (V)	Parameter *a*	Parameter *b*
2.5	337	0.7907
5	2854	0.8139
7.5	4371	0.8540
10	4211	0.8925

**Table 2:** Parameter estimates of Equation (11).

Voltage (V)	Parameter *c*	Parameter *d*
2.5	2.68	0.364
5	1.67	0.211
7.5	1.70	0.158
10	2.36	0.113

## Data Availability

The original contributions presented in this study are included in the article. Further inquiries can be directed to the corresponding author.
